# Preharvest and Postharvest Application of Garlic and Rosemary Essential Oils for Controlling Anthracnose and Quality Assessment of Strawberry Fruit During Cold Storage

**DOI:** 10.3389/fmicb.2020.01855

**Published:** 2020-08-14

**Authors:** Somaieh Hosseini, Jahanshir Amini, Mahmoud Koushesh Saba, Kaivan Karimi, Ilaria Pertot

**Affiliations:** ^1^Department of Plant Protection, College of Agriculture, University of Kurdistan, Sanandaj, Iran; ^2^Department of Horticultural Sciences, University of Kurdistan, Sanandaj, Iran; ^3^Safiabad Agricultural Research and Education and Natural Resources Center, Agricultural Research, Education and Extension Organization (AREEO), Dezful, Iran; ^4^Department of Sustainable Agro-Ecosystems and Bioresources, Research and Innovation Centre, Fondazione Edmund Mach (FEM), San Michele all’Adige, Italy; ^5^Center Agriculture Food Environment (C3A), University of Trento, San Michele all’Adige, Italy

**Keywords:** biofungicide, black spot, *Fragaria* × *ananassa* Duch, fruit quality parameters, volatile oils

## Abstract

This study assessed the feasibility of using essential oils (EOs) against *Colletotrichum nymphaeae* inciting strawberry anthracnose. Two EOs, extracted from *Allium sativum* (garlic) and *Rosmarinus officinalis* (rosemary), were selected because their fungicide efficacy was already well characterized under laboratory and greenhouse conditions. We characterized both EOs in terms of efficacy and impact on qualitative traits and sensory quality of strawberry fruit. The gas chromatography–mass spectrometry analysis confirmed the Diallyl trisulfide (29.08%) and (α)-pinene (15.779%) as the main components of *A. sativum* and *R. officinalis* EOs, respectively. Both *A. sativum* and *R. officinalis* EOs significantly inhibited the mycelial growth and conidial germination of *C. nymphaeae* in contact and vapor assays compared with untreated control. However, EC_50_ assay indicated *A. sativum* EO was more effective than *R. officinalis* EO against the pathogen. Malformations of the vegetative structures of the pathogen exposed to both EOs were revealed as shriveled, collapsed, and swelling mycelia in the cultures. Both EOs confirmed their efficacy under *in vivo* and greenhouse conditions; in fact, they significantly reduced the development of fruit decay and anthracnose disease incidence and severity, compared with untreated controls. Both EOs preserved sensory attributes and quality parameters of strawberry fruit including firmness, total soluble solids, ascorbic acid, antioxidant activity, and anthocyanin, but may leave unwanted smells. These findings suggest that two EOs can potentially represent an alternative to synthetic chemical fungicides against *C. nymphaeae* preserving fruit quality factors, although their cost and the impact on the fruit odor must be carefully taken into consideration before developing a commercial product.

## Introduction

Strawberry anthracnose caused by *Colletotrichum nymphaeae* (Pass.) Aa, is a major disease of strawberry (*Fragaria* × *ananassa* Duch.) limiting production worldwide by affecting fruits in preharvest and postharvest (Baroncelli et al., 2015; Karimi et al., 2017; Wang N.Y. et al., 2019). Although the pathogen causes symptoms on various parts of the plant, the most important damage is the fruit rot (Peres et al., 2005). In fact, large and sunken lesions on strawberry fruits make them unmarketable, leading to considerable production losses particularly under epidemic condition (Howard et al., 1992). Chemical fungicides, such as thiabendazole or azoxystrobin (Smith and Black, 1992; Wedge et al., 2007), are mainly used for the control of the disease, but their repeated application may result in serious problems including high residues on fruits, the development of fungicide resistance in pathogen populations, and environmental pollution (Karimi et al., 2016a). Furthermore, no suitable anthracnose-resistant strawberry cultivars have been obtained so far, mainly because resistant strawberry germplasms against the disease is not available. Considering the aforementioned concerns, a promising approach is the development of alternatives to synthetic chemical fungicides. In recent years, application of plant essential oils (EOs) received increasing attention as natural alternative to synthetic fungicides in the control of main postharvest diseases of different products (Sellamuthu et al., 2013; Fan et al., 2014; Ali et al., 2015; Shao et al., 2015; Vilaplana et al., 2018), including strawberries (Shao et al., 2013).

Essential oils are highly complex mixtures of volatile aromatic compounds synthesized by plants, which have diverse ecological functions, including the attraction of pollinators and defense mechanisms against harmful microorganisms (Bakkali et al., 2008; Medeiros et al., 2011; Li et al., 2013; Amaral et al., 2015; Costa et al., 2015; De Almeida et al., 2018). Monoterpenes, aldehydes, allyl phenols, alcohols, acids, and esters are the main components of plant EOs (Prakash et al., 2015; De Almeida et al., 2018). They are mainly produced and accumulated in undifferentiated cells, glandular trichomes, secretory ducts, and resin ducts (Nakatsu et al., 2000; Pimental et al., 2018).

Several studies have shown the inhibitory effect of plant EOs against phytopathogens (Bouchra et al., 2003; Daferera et al., 2003; Soylu et al., 2006; Bi et al., 2012; Ali et al., 2015; Amini et al., 2016). Some plant EOs have been evaluated against fruit rot of strawberry caused by *Rhizopus stolonifer*, *Penicillium digitatum*, *Aspergillus niger*, *Botrytis cinerea*, and *C. nymphaeae* (Reddy et al., 1998; Asghari et al., 2009; Nabigo and Morshedi, 2011; Zamani-Zadeh et al., 2013; Karimi et al., 2016a). Among the EOs studied so far, the antimicrobial activity of EOs from *Allium sativum* L. (garlic) (Corzomartinez et al., 2007; Mnayer et al., 2014; Satyal et al., 2017) and *Rosmarinus officinalis* L. (rosemary) (Mangena and Muyima, 1999; Soylu et al., 2006, 2010; Ozcan and Chalchat, 2008) has been proven against several plant pathogens and relatively well characterized. Some EOs may influence the quality of the treated products (Sivakumar and Bautista-Banos, 2014; Vilaplana et al., 2018). Although EOs may negatively affect the quality of fruits, for example, by giving a unwanted smell, there are no compressive studies on strawberry fruit rots, which take into consideration the fungicidal effect, their composition, and the side effect on fruit quality. In addition, to the best of our knowledge, inhibitory effects of *A. sativum* and *R. officinalis* EOs against *C. nymphaeae* were not yet assessed.

Therefore, the aim of this study was to evaluate the antifungal activity of two different EOs possessing a pungent odor, *A. sativum* and *R. officinalis*, against *C. nymphaeae*, under *in vitro*, *in vivo*, and greenhouse conditions and their effects on fruit quality parameters.

## Materials and Methods

### Pathogen

A highly pathogenic strain of *C. nymphaeae* (CCTUCCh32) provided from the culture collection of Tabriz University in Iran (Karimi et al., 2017) was used in the experiments. Fungal cultures were grown on potato dextrose agar (PDA; Merck Company, Germany) at 24°C ± 2°C for 10 days. Conidial suspension of *C. nymphaeae* was made by flooding fungal cultures with 15 mL of sterile distilled water containing Tween 80 (0.5%, vol/vol) and filtered through three layers of sterile cheesecloth to remove mycelial debris. The concentration of conidia was adjusted to 10^6^ spores/mL using hemocytometer.

### Fruits

Healthy and red ripened strawberry fruits (cv. Paros) having the same age and size were collected from an untreated strawberry farm in Kamyaran city, in Iran, at code 87 of BBCH (Biologische Bundesanstalt, Bundessortenamt und CHemische Industrie) scale (Meier, 2001). Fruits were transferred to laboratory, surface-disinfected with 70% ethanol for 30 s, rinsed with sterile distilled water (three times), and air-dried under a laminar flow hood for 10 min before further use.

### Essential Oil Extraction and Gas Chromatography–Mass Spectrometry Analysis

Essential oil of *A. sativum* was purchased from Zardband Pharmaceuticals Company, Tehran, Iran (no. 17-350-003). Essential oil of *R. officinalis* was extracted from aerial parts collected from a farm located in the University of Kurdistan, Iran, at flowering stage. Plant samples were air-dried in the dark at room temperature for 2 weeks and grounded, and EO was obtained by hydrodistillation using Clevenger-type distillation apparatus for 3 h. The resulting oil (approximately 1 mL of EO per 100 g of dried raw plant materials) was dried with anhydrous sodium sulfate and stored at 4°C in the dark in a glass bottle. Both EOs were analyzed with an Agilent 7890 A gas chromatography (GC) coupled with Agilent 5975C mass spectrometry (MS) (Agilent Technologies, United States), using an HP-5 MS capillary column (30 m × 0.25 mm, film thickness 0.25 μm). The analytical was carried out as described by Amini et al. (2016). Identification of the EOs components was performed by comparison of their retention indexes with those reported in the literatures (Ozcan et al., 2006) and their mass spectra with those in the WILEY/NBS.

### Minimal Inhibitory Concentration Using Contact and Vapor Assays

Six different concentrations of *A. sativum* (20, 155, 290, 430, 560, and 700 μL/L) and *R. officinalis* (100, 420, 740, 1,060, 1,380, and 1,700 μl/L) EOs were selected using a pretest analysis against *C. nymphaeae* (data not shown). The concentrations of EOs with 10 and 90% inhibition on the mycelial growth of the pathogen were subtracted and divided by five to determine other concentrations. The antifungal activity of the EOs was estimated as a reduction of the mycelial growth of *C. nymphaeae* under *in vitro* conditions using contact and vapor assays on PDA, described as follows. In contact assay, different amounts of EOs (μL/L) dissolved in 0.5% (vol/vol) Tween 80 were mixed to molten PDA to obtain the desired concentrations. The obtained solutions (60 mL) were added to each plate (9-cm diameter). Upon solidification, 5-day-old mycelial disk plug (5-mm diameter) of *C. nymphaeae* was placed in the center of each plate. Plates containing only freshly prepared PDA were used as control. The plates were incubated at 24°C ± 2°C for 10 days. Four plates were used per treatment. The percentage of inhibition was calculated using the following formula:

Inhibition percent = [(*d*_*c*_ − *d*_*t*_)/*d*_*c*_] × 100, where *d*_*c*_ is radial growth of the pathogen in control, and *d*_*t*_ is radial growth of pathogen in treatments (Thomidis and Filotheou, 2016).

In vapor assay, a sterile filter paper disk (5.0-mm diameter, Whatman no.1) dipped into different concentrations of EOs (μL/L) dissolved in 0.5% (vol/vol) Tween 80 was placed in the inner space of the inverted lid of the plates. One mycelial disk plug of the pathogen (5-mm diameter) was taken from the margin of a 5-day-old culture and was placed in the center of plate containing PDA. The plates were sealed with parafilm to avoid any loss of volatile compounds. The filter paper disks loaded with the sterile distilled water were used as control. Each treatment included four replicated plates. The plates were incubated at 24°C ± 2°C for 10 days. The percentage of inhibition was calculated using aforementioned formula. Finally, the concentrations of each EO (μL/L) providing 10% (EC_10_), 50% (EC_50_), and 90% inhibition (EC_90_) against *C. nymphaeae* were measured by interpolation from linear regression analysis using the SPSS v16.0 software.

Inhibitory effect of *A. sativum* and *R. officinalis* EOs on conidial germination of *C. nymphaeae* at selected concentrations (μL/L) was evaluated according to the method as described by Hosseni et al. (2020). For this purpose, 1 mL of different concentrations of each EO (μL/L) containing Tween 80 (0.5%, vol/vol) was pipetted into microtube (5 mL) containing 1 mL of the pathogen’s suspension at concentration of 10^6^ conidia/mL to obtain final concentrations. Microtubes were placed on the rotary shaker in 120 revolutions/min (rpm) at 25°C ± 2°C for 24 h. Afterward, 50 μL of resulting solutions was evenly spread on plates (9 cm) containing water agar medium (2% WA) using an L-shaped spreader. Essential oil-free tubes containing Tween 80 (0.5%, vol/vol) served as controls. Three replicates per treatment were prepared. Plates were incubated at 25°C ± 2°C for 48 h. For each replicate, 200 conidia were counted using an optical microscope (Olympus BX51, Japan; 40 × magnification using a micrometer), and the percentage of conidia germinated was calculated using the following formula:

Percentage of germinated conidia = number of conidia germinated/total number of conidia × 100.

A conidium was adverted to have germinated if the length of the germ tube was larger than or equal to the conidial diameter.

### Scanning Electron Microscopy

Total mycelial growth inhibition of the pathogen was observed with *A. sativum* and *R. officinalis* EOs at concentrations of 700 and 1,700 μL/L, respectively; therefore, the effect of EOs on the fungal structures of *C. nymphaeae* CCTUCCh32 was studied at a lower concentration (560 and 1,380 μL/L, respectively) using scanning electron microscopy (SEM) in contact assay (TESCAN MIRA3, Czechia). After 7 days, fungal structures of *C. nymphaeae* were freeze-dried, and morphological changes were observed.

### *in vivo* Antifungal Activity Assay

Effect of *A. sativum* and *R. officinalis* EOs on the development of fruit decay was studied using contact and vapor assays at concentrations of 700 and 1,700 μL/L, respectively. These selected concentrations were selected based on the total inhibitory effects under *in vitro* condition.

In contact assay, the fruits were dipped in sodium hypochlorite 1% for 1 min and then rinsed three times in sterile distilled water and let to dry under laminar hood on sterile filter paper. Dried fruits were treated with each EO in above given concentrations containing 0.5% (vol/vol) Tween 80 for 10 s. Treated and untreated fruits were then wounded (one mm wide and deep) using a sterilized scalpel at the fruit equator zone before inoculation. Conidial suspensions of *C. nymphaeae* (10^6^ conidia/mL, 10 μL) containing 0.5% (vol/vol) Tween 80 were then added to each wound on treated fruits. The wounded fruits inoculated with the pathogen’s inoculum and dipped only in sterile distilled water containing 0.5% (vol/vol) Tween 80 served as untreated inoculated and non-inoculated controls, respectively. Each treatment included four replicates containing five fruits each. The fruits were placed in plastic boxes under high relative humidity conditions (>80%) and incubated at 24°C ± 2°C with a 12-hour photoperiod. After 7 days, disease severity (DS) was measured using a scale of 0 to 5, where 0 represents healthy fruits, and 1, 2, 3, 4, and 5 represent <20%, 20.1% to 40%, 40.1% to 60%, 60.1% to 80%, and 80.1% to 100% of the fruit area rotted, respectively, as described by Huang et al. (2011).

In vapor assay, healthy strawberry fruits were disinfected as described above and dried under laminar hood. Each fruit was wounded (1 mm wide and deep) using a sterile scalpel. A conidial suspension of *C. nymphaeae* (10 μL) at concentration of 1.0 × 10^6^ conidia/mL containing 0.5% (vol/vol) Tween 80 was then added to each wound. Inoculated fruits were placed in plastic boxes and 2 mL of each EO was added to a plate (2-cm diameter and 2-cm height) and placed next to the fruits in the center of plastic box. Inoculated and non-inoculated fruits into the boxes containing sterile distilled water were determined as positive and negative controls. To avoid any loss of volatile compounds, the plastic boxes were immediately sealed with layers of parafilm and maintained at 24°C ± 2°C for 7 days. Each treatment included three replicates containing five fruits each. DS was measured as mentioned previously (Huang et al., 2011). The percentage of disease incidence (DI), disease severity (DS) and disease reduction (DR) were measured using the following formulae with some modifications (Nutter et al., 2006):

$$\mathrm{DI}\left(\%\right)=\frac{\mathrm{No}.\mathrm{of}\,\mathrm{infected}\,\mathrm{samples}\,\mathrm{per}\,\mathrm{treatement}}{\mathrm{Total}\,\mathrm{No}.\mathrm{of}\,\mathrm{assessed}\,\mathrm{samples}\,\mathrm{per}\,\mathrm{treatment}}\times 100$$

$$\mathrm{DS}\left(\%\right)=\frac{\sum \left(n\,i\times v\,i\right)}{N\times V}\times 100$$

Where *ni* is the number of samples with the same score, *vi* is the scoring index of DS from 0 to 5 corresponding to each sample, *N* is the total number of the samples, and *V* is the highest DS index score.

$$\mathrm{DR}\left(\%\right)=\frac{{\bar{\text{X}}}_{\text{c}}-{\bar{\text{X}}}_{\text{t}}}{{\bar{\text{X}}}_{\text{c}}}\times 100$$

Where ${\bar{\text{X}}}_{\text{t}}$ is the mean of DS per treatment, and ${\bar{\text{X}}}_{\text{c}}$ is the mean of DS in the untreated inoculated control.

### Greenhouse Assay

In the greenhouse trial, healthy 5-week-old strawberry plants (cv. Paros, susceptible to anthracnose fruit rot) were initially purchased at the beginning of September from a local strawberry nursery (Sanandaj, Iran) and transplanted into 10-cm-diameter pots containing clay/sand/manure mix in a ratio of 2:1:1. The pots were kept in greenhouse at 25°C ± 2°C, 60% to 70% RH, with a 16:8-h light–dark photoperiod and irrigated manually every 2 to 3 days. The plants were treated with *A. sativum* and *R. officinalis* EOs at concentrations of 700 and 1,700 μL/L EOs, respectively, until runoff using a handheld atomizer. After 24 h, treated plants were sprayed with a 5 mL conidial suspension of *C. nymphaeae* containing Tween 80 (0.5%, vol/vol) at concentration of 1.0 × 10^6^ conidia/mL and kept at 25°C ± 2°C, at high relative humidity (>80%) for 24 h covered with a 0.1-mm-thick transparent plastic film to preserve the moisture. The plants inoculated with fungal conidia and treated with distilled water containing Tween 80 (0.5%, vol/vol) were served as untreated non-inoculated and inoculated controls, respectively. Each treatment was composed of three replicates (three pots with one plant each), set up in a completely randomized block design. Disease severity was scored according to Delp and Milholland (1980), using a scale of 0 to 5, where 0 = “healthy petiole without lesions,” 1 = “petiole with lesions <3 mm in length,” 2 = “petiole with lesions from 3 to 10 mm,” 3 = “petiole with lesions from 10.1 to 20 mm,” 4 = “petiole with lesions from >20 mm,” and 5 = “entirely necrotic petiole and plant is dead.” Four randomly selected petioles per plant were assessed, and petiole infection by *C. nymphaeae* was confirmed by reisolation of the pathogen. The percentage of DI, DS, and DR were measured using aforementioned formula.

### Effect of EOs on the Postharvest Physicochemical Quality of Strawberry Fruit

In order to measure the effect of the EOs on the postharvest quality parameters of strawberry fruit, healthy fruits having the same age and size were treated separately with EOs of *A. sativum* and *R. officinalis* in a contact assay setup, at the selected concentrations of 700 and 1,700 μL/L, respectively. Fruits treated with distilled water were used as untreated control. Treated fruits were air-dried under a laminar flow hood for 10 min and stored into polyethylene bags in cold storage (1°C and 80–85% RH) for 8 days. Three replicates per treatment containing five fruits were examined. The quality parameters including weight loss (WL), firmness, total soluble solid (TSS), titratable acidity (TA), pH, ascorbic acid (AA), total flavonoid (TF), total phenol (TP), total antioxidant activity (TAA), peroxidase activity (POD), and anthocyanin were measured at harvest time (before treatment, 0 days) and 8 days after cold storage.

The strawberry fruits were weighed before treatment (M1) and after 8 days of storage (M2). WL was measured as [(M1 − M2)/M1] × 100 and expressed as percentage (Sogvar et al., 2016).

Fruit firmness was measured after 8 days of storage at two points on the equatorial region using a TA-XT2 texture analyzer (Santam, STM-1, Iran) fitted with an 8-mm-diameter cylindrical probe. The probe descended toward the sample at the speed of 0.5 mm s^–1^. The firmness was defined as the maximum force (N).

Two pieces on two opposite sides of each fruit were isolated, pooled, and juiced for TSS and TA analyses. The TSS was recorded at harvest time and after 8 days of storage using a temperature-compensated refract meter (Atago ATC Co., Japan). The TA was recorded by titrating 3 mL of juice in 27 mL distilled water with 0.1 N NaOH solutions to pH 8.1. The TA content was defined as the percentage of citric acid (%). The pH of fruit juice was recorded using a pH meter (Motorhome, Switzerland).

AA was determined for each treatment according to the method described by Kevers et al. (2007). Two pieces on two opposite sides of each fruit were frozen in liquid nitrogen and conserved at −80°C until used for study. Frozen fruit sample (0.5 g) was added to 1 mL of 0.5% metaphosphoric acid (wt/vol) and was shaken at 12,000 rpm for 15 min at 4°C. The reaction mixture absorption was measured with spectrophotometer (Unico UV 2100, United States) at 510 nm. The results were expressed as mg AA per 100 g of fruit fresh weight (FW) using aqueous AA standard (Koushesh Saba and Moradi, 2016).

The TF in strawberry fruit was measured using the method described by Zhishen et al. (1999) with some modifications. Two hundred microliters of fruit extracts was dissolved in 1,280 μL distilled water and 60 μL 5% NaNO_2_ (wt/vol). After 5 min, 60 μL 10% AlCl_3_ (wt/vol) and 40 μL NaOH (1 mol L^–1^) were added to the mixture, respectively. The final mixture was stirred, and absorbance was recorded at 510 nm. The results were expressed as mg of rutin per 100 g of fruit FW.

The TP of fruit juice was evaluated using the method described by Singleton et al. (1999) with some modifications. Briefly, 20 μL fruit extracts was added to 250 μL distilled water, 750 μL solution of 10% Folin–Ciocâlteu (vol/vol), and 800 μL 7.5% Na_2_CO_3_ (wt/vol). Samples were placed in darkness for 1 h at 25°C. The absorbance of the samples was read at 765 nm using spectrophotometer. The TP was reported as mg gallic acid equivalents per 100 g fruit FW using aqueous gallic acid standard.

The TAA was measured using 2,2-diphenyl-1-picrylhydrazyl (DPPH) assay as described by Patras et al. (2009). For measurement of antioxidant activity, 200 μL of fruit crude extracts was mixed with 500 μL DPPH (0.238 mg per 1 mL methanol). After 30 min at 30°C, absorbance of the samples was recorded at 515 nm, and radical scavenging activity was calculated using the following formula:

Percentage of DPPH inhibition (IP) was measured as follows:

IP = [(*A*_*b*_ − *A*_*s*_)/*A*_*b*_) × 100, where *A*_*b*_ and *A*_*s*_ are the absorbance values of the control and treatment, respectively.

The POD activity was tested using the method described by Ghanati et al. (2002) with some modifications. Approximately 0.5 g frozen fruit powder was ground with 2 mL cold 100 mmol L^–1^ phosphate-buffered sodium (pH 7) containing 0.025 g polyvinylpolypyrrolidone (wt/vol). The homogenate was centrifuged at 15,000 rpm at 4°C for 15 min. One milliliter of resulting supernatants was used as crude enzyme extracts for assaying the enzyme activities. Four hundred microliters of crude enzyme extract was dissolved in 400 μL 50 mmol L^–1^ phosphate-buffered potassium (pH 6.6), 140 μL 0.03% H_2_O_2_, and 140 μL 1% guaiacol. The unit of enzyme activity was expressed as an increase in absorbance at 470 nm at 25°C for 2 min.

The anthocyanin content of fruit juice was measured using a pH difference as described by Cheng and Breen (1991). Using two buffers with different pHs including 400 mL 1 mol CH_3_COONa, 240 mL 1 N HCl, 360 mL distilled water, pH 4.5, and 125 mL 0.2 N KCl, 385 mL 0.2 N HCl, pH 1. In continue 1 g of strawberry frozen tissue was added to 8 mL buffers separately and homogenized. The homogenate was then centrifuged at 6,000 rpm at 4°C for 15 min. The absorbance of the supernatants was monitored at 520 and 700 nm at two pH values (1 and 4.5) using spectrophotometer (Unico UV 2100, United States). The following formula was used to calculate anthocyanin content based on pelargonidin triglycoside: *A* = (*A*520 nm − *A*700 nm) pH 1 − (*A*520 nm − *A*700 nm) pH 4.5.

Total anthocyanin (mg/L) = (*A* × 433.1 × 10^3^ × DF)/ (22,400 × 1 × 1,000), where DF = dilution factor, 433.1 = molecular weight of pelargonidin, and 22,400 = molar extinction coefficient, L mol^–1^ cm^–1^.

### Sensory Analysis

The sensory analysis was carried out according to Lu et al. (2017) with some modifications. It was carried out in a sensor panel room at the University of Kurdistan in three different days at 10:00 AM at 25 to 29°C. Eleven trained panelists (assessors, five males and six females, aged 20–30 years) on a 5-point scale (1 = very weak, 2 = weak, 3 = intermediate, 4 = good, and 5 = very good) for the sensory parameters including flavors, odor, taste (sweet, sour), and overall acceptance were studied. All parameters were measured at harvest time and 8 days of storage. There was 5-min interval between testing with providing water between assessors.

### Statistical Analysis

Parametric data in this study were statistically analyzed as a completely randomized design by standard analysis of variance after verification of normal distribution and homoscedasticity using SAS version 8.2 software. The means were compared with Tukey test and presented as mean ± standard error.

The data for sensory evaluation were tested for normality, and because of lacking normality distribution, all data were subjected to non-parametric analysis using Kruskal–Wallis test by SPSS (Statistical Product and Service Solutions) version 16.0.

## Results

### Chemical Composition of the EOs

The chemical compositions of the *A. sativum* and *R. officinalis* EOs were revealed by GC-MS, and the results are presented in Tables 1, 2. A total of 8 and 19 compounds were identified in the *A. sativum* and *R. officinalis* EOs, representing 99.27 and 98.56% of the total oil compositions, respectively. The major components of *A. sativum* EO were allyl trisulfide (29.08%), diallyl disulfide (28.23%), and diallyl sulfide (16.106%), whereas α-pinene (15.779%), bornyl acetate (12.660%), camphor (11.998%), and 1,8-cineol (11.268%) constituted the most abundant compounds of *R. officinalis* EO.

**TABLE 1:** Chemical composition of the *Allium sativum* L. essential oil.

**Number**	**Compound**	**Molecular formula**	**CAS no.**	**Area (%)**	**Retention time (min)**
1	1-Propene, 3,3′-thiobis [diallyl sulfide]	C_6_H_10_S	592-88-1	16.106	3.646
2	Diallyl disulfide	C_6_H_10_S_2_	2179-57-9	28.225	6.100
3	Acetic acid, 2-(thiocarboxy) hydrazide, *O*-methyl ester	C_4_H_8_N_2_O_2_S	20184-99-0	4.981	6.192
4	3-Vinyl-1,2-dithiacyclohex-5-ene	C_6_H_8_S_2_	62488-53-3	1.501	7.457
5	Trisulfide, di-2-propenyl [allyl trisulfide]	C_6_H_10_S_3_	2050-87-5	29.802	8.429
6	1,2,4-Thiadiazol-5-amine, 3-methyl [CTK8C1752]	C_3_H_5_N_3_S	17467-35-5	7.659	8.504
7	Acetic acid,3-chlorophenyl ester [3-chlorophenol acetate]	C_8_H_7_ClO_2_	13031-39-5	2.155	9.076
8	Tetrasulfide, di-2-propenyl [diallyl tetrasulfide]	C_6_H_10_S_4_	2444-49-7	9.571	10.873
Total	—	—	—	99.27	−

**TABLE 2:** Chemical composition of the *Rosmarinus officinalis* L. essential oil.

**Number**	**Compound**	**Molecular formula**	**CAS no.**	**Area (%)**	**Retention time (min)**
1	1R. alpha.-pinene [α-pinene]	C_10_H_16_	7785-70-8	15.779	4.501
2	Camphene	C_10_H_16_	79-92-5	7.199	4.649
3	3-Octanone	C_8_H_16_O	106-68-3	1.152	4.970
4	Bicyclo[3.1.1]heptane, 6,6-dimethyl-2-methylene, (1S)-[β-pinene]	C_10_H_16_	18172-67-3	4.223	5.021
5	*trans*-3-Caren-2-ol	C_10_H_16_O	93905-79-4	2.276	5.456
6	Eucalyptol [1,8-cineol]	C_10_H_18_O	470-82-6	11.268	5.565
7	Cyclohexene, 1-methyl-4-(1-methylethylidene)-	C_10_H_16_	586-62-9	0.817	6.091
8	1,6-Octadien-3-ol, 3,7-dimethyl-[linalool]	C_10_H_18_O	78-70-6	3.787	6.280
9	Bicyclo[2.2.1]heptan-2-one, 1,7,7-trimethyl, (1R)-[camphor]	C_10_H_16_O	464-49-3	11.988	6.852
10	Bicyclo[3.1.1]heptan-3-one, 2,6,6-trimethyl, (1.alpha.,2.alpha.,5.alpha.)-[pinocamphone]	C_10_H_16_O	547-60-4	1.211	6.944
11	Borneol	C_10_H_18_O	10385-78-1	7.448	7.087
12	3-Cyclohexen-1-ol, 4-methyl-1-(1-methylethyl)-[terpinen-4-ol]	C_10_H_18_O	562-74-3	2.401	7.133
13	3-Cyclohexene-1-methanol,.alpha.,.alpha.4-trimethyl-[α-terpinol]	C_10_H_18_O	98-55-5	2.350	7.299
14	3-Cyclopentene-1-ethanol, 2,2,4-trimethyl-	C_10_H_18_O	10385-78-1	1.496	7.447
15	Bicyclo[3.1.1]hept-3-en-2-one, 4,6,6-trimethyl-[D-verbenone]	C_10_H_14_O	80-57-9	7.027	7.550
16	Cyclohexanol, 2-methyl-5-(1-methylethenyl)-[neodihydrocarveol]	C_10_H_18_O	619-01-2	1.305	7.785
17	Ethanol, 2-(3,3-dimethylcyclohexylidene), (Z)-	C_10_H_18_O	26532-23-0	1.621	7.859
18	Bicyclo[2.2.1]heptan-2-ol, 1,7,7-trimethyl, acetate, (1S-endo)-[bornyl acetate]	C_12_H_20_O_2_	5655-61-8	12.660	8.266
19	Caryophyllene	C_15_H_24_	87-44-5	2.558	9.587
Total	—	—	—	98.567	—

### *In vitro* Antifungal Activity

**FIGURE 1:**
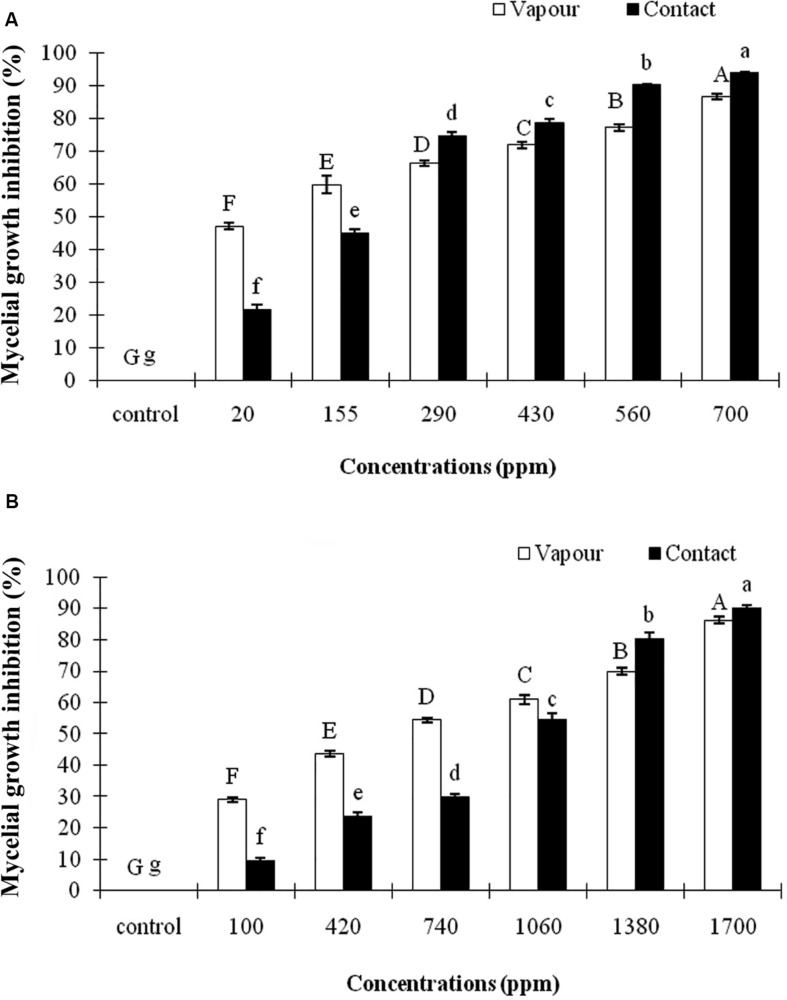
Inhibitory effect of different concentrations of *Allium sativum* L. **(A)** and *Rosmarinus officinalis* L. **(B)** essential oils on the mycelial growth of *Colletotrichum nymphaeae* CCTUCCh32 on potato dextrose agar based on contact and vapor assays. Different letters show significant difference (*P* ≤ 0.01) according to Tukey test.

In this study, both EOs of *A. sativum* and *R. officinalis* significantly (*p* ≤ 0.01) inhibited the mycelial growth of *C. nymphaeae* in contact and vapor phases under *in vitro* compared with control. The inhibitory efficacy increased concomitantly with an increase in EOs concentration (Figure 1). Moreover, the vapor effect of both EOs was found to be more effective in the reduction of the mycelial growth of *C. nymphaeae* compared with contact effect (Table 3). Both EOs in this study exhibited varying degrees of antifungal activity against *C. nymphaeae*. However, the present data showed that *A. sativum* EO was more toxic than *R. officinalis* EO to *C. nymphaeae* at the same dosage (Table 3). Overall, the EO of *A. sativum* (EC_50_10.10 and 3.74 μL/L) exhibited significantly higher antifungal activity than *R. officinalis* EO (EC_50_ 75.49 and 43.77 μL/L) against pathogen in both contact and vapor assays (Table 3).

**TABLE 3:** Comparisons of MIC_10_, MIC_50_, and MIC_90_ of *Allium sativum* L. and *Rosmarinus officinalis* L. essential oil (μL/L) in reduction of the mycelial growth of *Colletotrichum nymphaeae* CCTUCCh32 (at confidence limit 95%) using contact and vapor assays on potato dextrose agar.

**Plant EOs**	**MIC_10_**	**MIC_50_**	**MIC_90_**	***a***	***b*^2^**	χ**^2^**	***R*^2^**
	**Contact method**						

*A. sativum*	1.28	10.10	79.57	1.43 ± 0.11	−1.43 ± 0.16	17.88	0.90
*R. officinalis*	18.56	75.49	306.96	2.11 ± 0.17	−3.96 ± 0.34	45.56	0.81

	**Vapor method**						

*A. sativum*	0.03	3.74	421.24	0.62 ± 0.11	−0.35 ± 0.14	7.35	0.81
*R. officinalis*	3.06	43.77	624.42	1.11 ± 0.13	−1.82 ± 0.24	12.63	0.83

*MIC_10_, MIC_50_, and MIC_90_ represent minimum inhibitory concentration of essential oil to inhibit mycelial growth to 10, 50, and 90%, respectively. *a* = Slope; *b*^2^ = intercept; *R*^2^ = correlation coefficient.*

**FIGURE 2:**
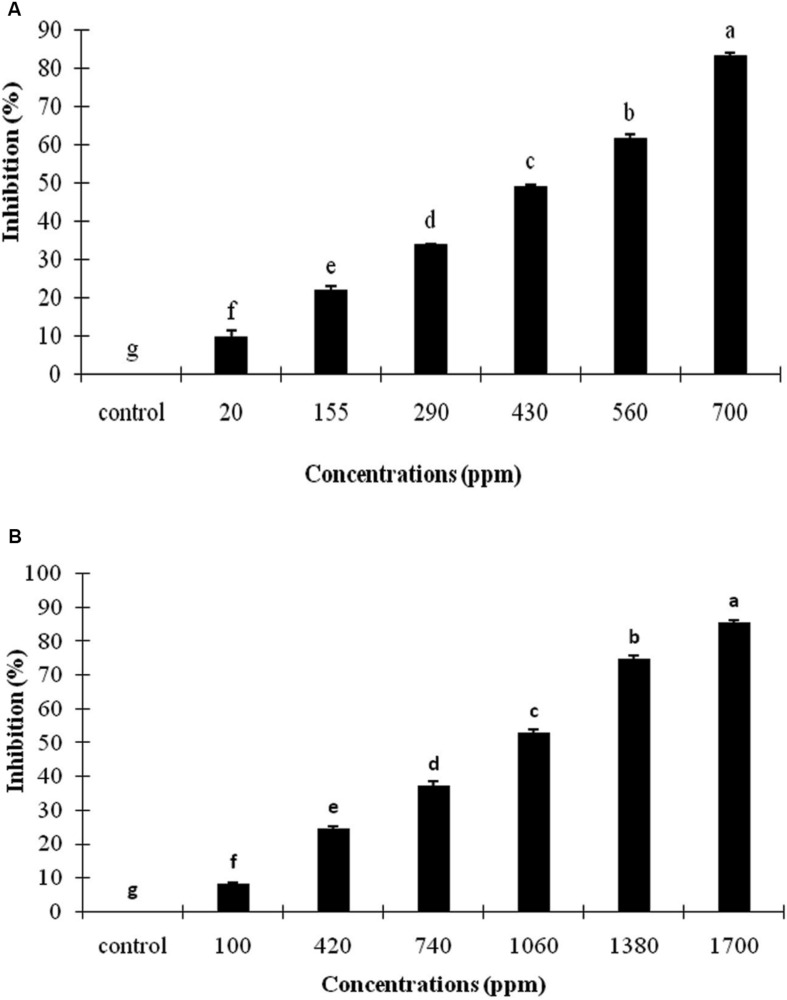
Inhibitory effect of *Allium sativum* L. **(A)** and *Rosmarinus officinalis* L. **(B)** essential oils on conidial germination of *Colletotrichum nymphaeae* CCTUC Ch32 under *in vitro*. Means with different letters show significant difference (*P* ≤ 0.01) according to Tukey test.

The effects of *A. sativum* and *R. officinalis* EOs on the conidial germination of *C. nymphaeae* are presented in Figure 2. The conidial germination of *C. nymphaeae* was significantly inhibited by both EOs at selected concentrations, and inhibition rate was increased concomitant with an increase in EOs concentration (Figure 2).

### Effect of Plant EOs on the Hyphae Morphology

**FIGURE 3:**
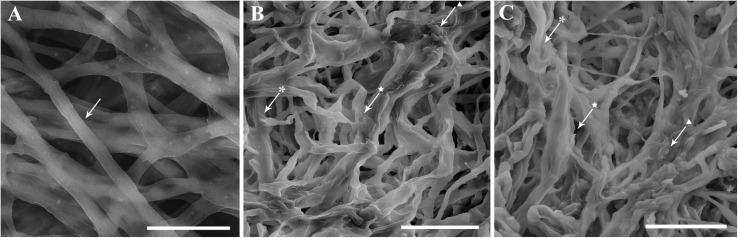
Morphological responses of *Colletotrichum nymphaeae* CCTUCCh32 to the effects of *Allium sativum* L. **(B)** and *Rosmarinus officinalis* L. **(C)** essential oils compared with untreated control **(A)** revealed by SEM images. Arrows show obvious malformations as shriveled (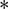), collapsed (★), and swelling (▲) mycelia of *C. nymphaeae* exposed to *A. sativum* and *R. officinalis* essential oils compared with intact mycelial structures in untreated control. Scale bars: A = 10 μm, B and C = 20 μm.

Morphological malformations in the fungal structures of *C. nymphaeae* exposed to *A. sativum* and *R. officinalis* EOs were observed using SEM (Figure 3). Fungal structures of *C. nymphaeae* showed classical degenerative changes as shriveled, collapsed, and swelling mycelia showing also cytoplasmic coagulation, which were not present in untreated control (Figure 3).

### Efficacy of EOs on Fruit Decay Development Under *in vivo*

The efficacy of the EOs to reduce fruit decay development in both contact and vapor assays is presented in Table 4. The DI was 100% in the untreated inoculated control, in both contact and vapor assays. Anthracnose symptoms in untreated inoculated fruits were observed as black, sunken necrotic lesions containing conidial mass on fruits (data not shown). On the contrary, the percentage of DI in the inoculated fruits treated with EOs of *A. sativum* and *R. officinalis* in contact phase was 37.5 and 62.5%, respectively, whereas the corresponding results in the vapor assay were 12.5 and 6.25%, respectively. Moreover, both EOs significantly (*p* ≤ 0.01) reduced the severity of fruit decay development compared with untreated inoculated control in both contact and vapor assays. In contact phase, *A. sativum* and *R. officinalis* EOs reduced the fruit decay development by 90.62 and 84.37%, respectively, whereas the corresponding results were 97.36% (*A. sativum*) and 98.68% (*R. officinalis*) in the vapor assay without any significant difference with untreated non-inoculated control (Table 4).

**TABLE 4:** Effect of *Allium sativum* L. and *Rosmarinus officinalis* L. essential oils in reduction of fruit decay development on strawberry fruits (cv. Paros) caused by *Colletotrichum nymphaeae* CCTUCCh32, 7 days after inoculation under *in vivo* condition.

**Treatments**	**Contact method**			**Vapor method**		
	**DS (%)**	**DR (%)**	**DI (%)**	**DS (%)**	**DR (%)**	**DI (%)**
Inoculated control	80 ± 2.04*a*	n.a.*	100	95 ± 2.04*a*	n.a.*	100
*A. sativum* 700 μL/L	7.5 ± 2.5*b*	90.62	37.5	2.5 ± 1.44*b*	97.36	12.5
*R. officinalis* 1,700 μL/L	12.5 ± 1.44*b*	84.37	62.5	1.25 ± 1.25*b*	98.68	6.25
Untreated non-inoculated control	0.00 ± 00*c*	n.a.*	0.00	0.00 ± 00*b*	n.a.*	0.00

Means ± standard error in the same column with different letters are significantly different (*P* ≤ 0.05) according to Tukey test. *Not applicable. DS, disease severity; DR, disease reduction; DI, disease incidence.

### Efficacy of EOs on Disease Severity of Strawberry Anthracnose in Greenhouse

Disease symptoms were first observed on petioles in the untreated non-inoculated treatment 35 days after inoculation under greenhouse condition. Disease severity was scored 60 days after inoculation with the pathogen, corresponding to the highest level of DS in the untreated non-inoculated control. Disease incidence and severity scoring indicates that inoculated strawberry plants treated with *A. sativum* and *R. officinalis* EOs had highly significantly (*p* ≤ 0.01) lower disease compared to untreated non-inoculated control (Table 5). *Allium sativum* EO showed the highest efficacy in DS reduction (96.15%) at the concentration of 700 μL/L followed by *R. officinalis* EO with 76.92% at the concentration of 1,700 μL/L compared with untreated inoculated control (Table 5). Disease severity in treatments treated with *A. sativum* EO was not significantly different to untreated non-inoculated control (Table 5).

**TABLE 5:** Effect of *Allium sativum* L. and *Rosmarinus officinalis* L. essential oils on the reduction of anthracnose symptoms on strawberry plants (cv. Paros) in greenhouse conditions after 2 months of inoculation with *Colletotrichum nymphaeae* CCTUCCh32.

**Treatments**	**DS (%)**	**DR (%)**	**DI (%)**
Inoculated control	86.664.4a	n.a.*	100
*A. sativum* 700 μL/L	3.331.66c	96.15	16.66
*R. officinalis* 1,700 μL/L	202.88b	76.92	83.33
Untreated non-inoculated control	0.000.00c	n.a.*	0.00

Means ± standard error in the same column with different letters are significantly different (*P* ≤ 0.05) according to Tukey test. *Not applicable. DS, disease severity; DR, disease reduction; DI, disease incidence.

### Efficacy of EOs on Fruit Quality

No difference was detected between untreated control and the fruit treated with EOs in terms of WL. The WL rate of *R. officinalis* and *A. sativum* EOs after 8 days of storage was 0.50 and 0.32%, respectively, whereas WL for untreated control was 0.41% (Table 6).

**TABLE 6:** Effect of *Allium sativum* L. and *Rosmarinus officinalis* L. essential oils at concentrations of 1,700 and 700 μL/L, respectively on strawberry fruit quality after 8 days’ storage.

**Paros cultivar**	**Treatments**	**Spray time**	**D8**
WL (%)	Control	00.00b	0.410.14a
	*R. officinalis*		0.500.16a
	*A. sativum*		0.320.02a
Firmness (N)	Control	4.60.49a	3.200.15a
	*R. officinalis*		4.20.14a
	*A. sativum*		4.20.25a
pH	Control	3.470.03b	4.100.06a
	*R. officinalis*		3.790.02a
	*A. sativum*		3.830.01a
TA (%)	Control	1.120.07a	0.730.05b
	*R. officinalis*		0.830.01b
	*A. sativum*		0.810.02b
TSS (g/100 g	Control	8.50.66b	10.50.41a
FW)	*R. officinalis*		8.560.43b
	*A. sativum*		9.33.0.56 ab
AA (mg/100 g	Control	25.592.78a	21.133.01a
FW)	*R. officinalis*		22.961.41a
	*A. sativum*		24.131.11a
TF (mg/100 g	Control	132.706.83a	127.462.77*ab*
FW)	*R. officinalis*		119.447.23*ab*
	*A. sativum*		114.441.40b
TP (mg/100 g	Control	156.3220.27b	219.968.88a
FW)	*R. officinalis*		199.928.72*ab*
	*A. sativum*		206.7811.35a
Antioxidant	Control	76.731.83a	71.040.26b
(DPPH%)	*R. officinalis*		81.131.80a
	*A. sativum*		77.161.43a
Anthocyanin	Control	13.122.06a	14.212.01a
(mg/100 g FW)	*R. officinalis*		17.763.75a
	*A. sativum*		17.060.98a
POD	Control	0.0940.06*c*	0.600.03a
(U min^–1^ g^–1^ FW)	*R. officinalis*		0.450.07b
	*A. sativum*		0.620.02a

*Means ± standard error with different letters are significantly different (*P* ≤ 0.05) according to Tukey test.*

Fruit firmness gradually decreased during storage, and no significant differences were observed between treatments. However, the fruits treated with both EOs showed higher firmness than untreated fruits after storage. Firmness of the fruits treated with both EOs and untreated control was 4.20 and 3.20 at the end of storage (Table 6).

During storage, the pH of strawberry fruits increased, whereas TA decreased. However, no significant difference was observed between treated and untreated fruits in terms of pH and TA (Table 6).

The TSS content in untreated fruit was 8.5% at harvest time and increased to 10.5% at the end of storage, whereas the fruits treated with *R. officinalis* EO significantly (*p* ≤ 0.05) preserved the TSS content during storage compared with untreated control (Table 6).

The AA rate was reduced in all treatments after 8 days of the storage, but there was no significant difference between the treatments. The AA content of strawberry fruits in untreated control at harvest time and end of storage was 25.59 and 21.13 mg/100 g FW, respectively (Table 6).

In this study, the TF was reduced in all treatments at the end of storage. *Allium sativum* EO (114 mg/100 g FW) showed the lower TF than untreated control (127.46 mg/100 g FW) at the end of storage (Table 6). The TP rate of fruits in all treatments increased during storage, but no significant differences were detected between treatments after storage. The TP of fruits in untreated control was 156.32 and 219.96 mg/100 g FW at harvest time and end of storage, respectively (Table 6). The results of the antioxidant assays indicated that DPPH values of fruits treated with *A. sativum* EO (77.16%) and *R. officinalis* (81.13%) were significantly (*p* ≤ 0.05) higher than untreated control (71.04%) at the end of storage (Table 6). The DPPH of the fruits in untreated control was decreased at the end of storage (Table 6).

Anthocyanin rate increased in all treatments treated with *A. sativum* and *R. officinalis* EOs during storage, but without any significant difference with untreated control. However, the anthocyanin rate of fruit treated with *A. sativum* EO (17.06 mg/100 g FW) and *R. officinalis* EO (17.76 mg/100 g FW) was higher than untreated control (14.21 mg/100 g FW) (Table 6).

The POD in all treatments increased from the beginning until the eighth day of cold storage. The POD of untreated control and the fruits treated with *R. officinalis* and *A. sativum* EOs was 0.6, 0.45, and 0.62 U min^–1^ g^–1^ FW, respectively, at the end of storage (Table 6). Our results showed that POD activity of strawberry fruits treated with of *R. officinalis* EO was significantly (*p* ≤ 0.05) less than untreated control at the end of storage.

### Sensory Evaluation

**FIGURE 4:**
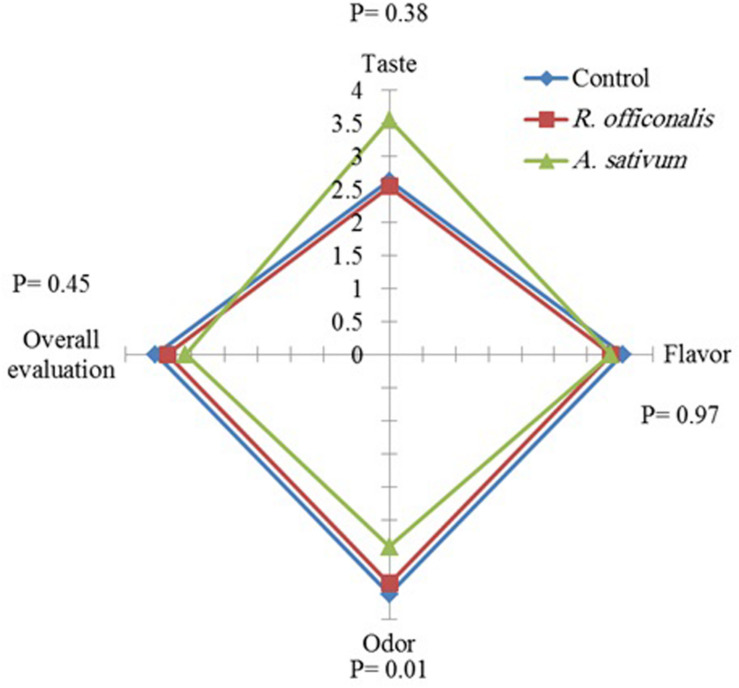
Influence of *Allium sativum* L. and *Rosmarinus officinalis* L. essential oils on sensory quality attributes of strawberry fruit (cv. Paros) after 8 days’ storage at 0°C.

The sensory analysis of the fruits treated with EOs did not reveal any adverse effect on fruit taste, flavor and overall evaluation significantly, but the odor was altered. The odor of the fruits treated with *A. sativum* EO was significantly (*p* ≤ 0.05) affected and categorized as the lowest rank based on sensory evaluation, whereas no significant difference was found between *R. officinalis* EO and untreated control (Figure 4).

## Discussion

During the past decades, many efforts have been made to find eco-friendly alternatives, such as EOs derived from medicinal plants, to substitute synthetic chemical fungicides for the management of postharvest pathogens. *Colletotrichum nymphaeae* that causes strawberry anthracnose is, together with *B. cinerea*, among the most dangerous postharvest pathogens of strawberry (Reddy et al., 1998; Huang et al., 2011; Karimi et al., 2017; Wang N.Y. et al., 2019). The feasibility of using EOs as fungicides to control *C. nymphaeae* is poorly studied. Among the most studied and effective EOs, we selected *A. sativum* and *R. officinalis* EOs, and we analyzed chemical composition, antifungal activities, and their effect on the postharvest quality of strawberry fruits during storage period, with the aim of assessing the feasibility of using EOs as eco-friendly disease control measure.

We confirmed that the EO chemical composition is in line with what was already reported in literature. In fact, the analysis of *A. sativum* EOs revealed the sulfur-containing compounds including diallyl disulfide, allyl trisulfide, and diallyl tetrasulfide as the major compounds of *A. sativum* EO, and the related concentration within the range reported in previous studies (Kim et al., 2004; Corzomartinez et al., 2007; Mnayer et al., 2014; Satyal et al., 2017; Wang Y. et al., 2019). The *A. sativum* EO is, therefore dominated by allyl polysulfides. These compounds are well known for their antifungal, antibacterial, acaricidal, antiparasitic, nematicidal, antiviral, and insecticidal properties (Kim et al., 2004; Amini et al., 2015; Satyal et al., 2017; Wang Y. et al., 2019). On the contrary, monoterpenes and oxygenated monoterpenoids constituted the most abundant compounds of *R. officinalis* EO (Table 2). The abundance of monoterpenes and monoterpenoid compounds obtained from *R. officinalis* EO in this study was in the range reported by previous investigations (Bouchra et al., 2003; Ozcan and Chalchat, 2008; Pitarokili et al., 2008; Laborda et al., 2013; Barreto et al., 2014; da Silva Bomfim et al., 2015; Bajalan et al., 2017; Abada et al., 2020). However, the range of relative concentrations of each single component is quite variable in literature. The observed differences might be due to several factors, such as differences in place and cultivation conditions, plant species, soil type, harvest time, climatic and seasonal factors, and geographical origins (Hajlaoui et al., 2010; Rahimi-Nasrabadi et al., 2013; Bitu et al., 2015; Grulova et al., 2015; Lee et al., 2015; Alencar-Filho et al., 2017). The high variability poses an important question on how to standardize the composition of the EOs for the use as plant protection products, in particular under regulations, as in the European Union, where the consistency in the composition is a prerequisite in the registration process (EU Regulation 1107/2009). The high variability in the composition makes it also very difficult to understand which component(s) and/or their relative concentration in the mixture is responsible for the fungicide efficacy. In addition, most of the studies on the single components focus on the major ones, and the role in the antifungal activity of the minor components in the EO composition is, consequently, most of the time neglected.

Antifungal activity of *R. officinalis* EO and its monoterpenes compounds has been well documented against different phytopathogens including *Alternaria alternata*, *B. cinerea*, *Sclerotinia sclerotiorum, Phytophthora nicotianae, Sclerotium cepivorum, Fusarium oxysporum* f. sp. *dianthi*, and *F. proliferatum* (Bouchra et al., 2003; Ozcan and Chalchat, 2008; Pitarokili et al., 2008), although efficacy is noticed also against insects (i.e., *Ectomyelois ceratoniae*), mites (i.e., *Tetranychus urticae*), bacteria (i.e., *Streptococcus agalactiae*, *Staphylococcus aureus*, *Escherichia coli*, *Klebsiella pneumoniae*, *Listeria monocytogenes*, and *Bacillus subtilis*), and yeasts (i.e., *Candida* strains) (Laborda et al., 2013; Barreto et al., 2014; da Silva Bomfim et al., 2015; Bajalan et al., 2017; Abada et al., 2020).

As expected from the results reported in aforementioned studies, *A. sativum* and *R. officinalis* EOs could inhibit the mycelial growth of the *C. nymphaeae* in contact and vapor phases under *in vitro* conditions, although the highest inhibitory activity was observed in vapor phase compared with contact phase (Table 3). This result was also in line with the study of Soylu et al. (2010), where the effect of the EOs of *Origanum syriacum*, *Lavandula stoechas*, and *R. officinalis* against *B. cinerea* in vapor phase was higher than contact phase. It could be due to the better attachment of EO molecules to the lipophilic fungal mycelia in vapor phase, exerting antifungal effect directly on the mycelia (Ali et al., 2015). Moreover, these EOs could inhibit the conidial germination of *C. nymphaeae* at selected concentrations, although inhibition potential of *A. sativum* EO appeared at lower concentrations compared with *R. officinalis* EO (Figure 2). In some studies, the influence of dill, lemongrass, and cinnamon EOs on mycelial growth and conidial germination reduction of *C. nymphaeae*, *Colletotrichum gloeosporioides*, and *Colletotrichum musae* has been well shown (Maqbool et al., 2011; Karimi et al., 2016b). However, suppression of conidial germination is most probably related to destructive effects of EOs presented here on cell wall and permeability of plasma membrane of the conidia as observed for vegetative structures of the pathogen using SEM observations. Distinctive changes in vegetative structures of *C. nymphaeae* cultures exposed to both *A. sativum* and *R. officinalis* EOs were revealed as shriveled, collapsed, and swelling mycelia together with cytoplasmic coagulation (Figure 3); these observations were in line with other studies, where the mycelium of *C. nymphaeae* and *B. cinerea* exposed to dill and Syrian oregano EOs showed considerable morphological alterations in their vegetative structures compared with untreated control (Soylu et al., 2010; Karimi et al., 2016a). Morphological damage to fungal hyphae in this study might be due to leakage of ions and metabolites caused by altered membrane permeability and the breaking of fungal cell wall (Bordoh et al., 2020).

Similarly to *in vitro* tests, the strawberry fruits treated with both *A. sativum* and *R. officinalis* EOs in volatile phase exhibited the less DS compared with contact phase (Table 4). This result highlights the more toxicity of volatile compounds of both *A. sativum* and *R. officinalis* EOs than those that are toxic in contact phase. In an accordant study, strawberry fruits treated with *Anethum graveolens* EO showed the less DS caused by *C. nymphaeae* compared with untreated control (Karimi et al., 2016b). In other studies, the *R. officinalis* EOs have been shown to be effective in reducing postharvest decay of table grapes, stone, and pome fruit (Lopez-Reyes et al., 2010, 2013; Servili et al., 2017). Furthermore, our findings confirmed the results of previous studies describing the antifungal activity of *A. sativum* against *B. cinerea* and *Penicillium expansum* as the causal agent of fruit decay in citrus and apples (Obagwu and Korsten, 2003; Daniel et al., 2015).

Under greenhouse condition, at the tested dosages, *A. sativum* EO had higher efficacy in the reduction of anthracnose DI and severity than *R. officinalis* EO without any significant difference with untreated non-inoculated control (Table 5). This result was in line with *in vivo* and *in vitro* tests. In general and unlike *in vitro* assays, very few studies have been conducted under greenhouse condition to assess antifungal activity of EOs against plant pathogens (Amini et al., 2015, 2016; La Torre et al., 2016; Hosseni et al., 2020). The effect of plant EOs as volatile compounds on the surface of strawberry fruits and leaves may have induced a stressful environment, which could trigger phenolic compounds and peroxidase synthesis, creating a defense system in strawberry plant against phytopathogens (Ben-Jabeur et al., 2015). Furthermore, the phenolic compounds in the EOs have been proven to prevent the mycelial growth of *Alternaria citri* as the causal agent of orange black rot and reduce the severity of decay in orange as well (Ramezanian et al., 2016). Application of plant EOs against soil-borne pathogens (Amini et al., 2015, 2016; La Torre et al., 2016), foliar diseases (Hong et al., 2015; Jing et al., 2018), and postharvest diseases (Thomidis and Filotheou, 2016; Servili et al., 2017) has been studied.

In this study, *A. sativum* and *R. officinalis* EOs did not reduce water loss and delayed the qualitative changes in fruit firmness, PH, AA, TA, TP, TF, and anthocyanin, compared with untreated control (Table 6). On the contrary, a significant difference was found between the strawberry fruits treated with *A. sativum* and *R. officinalis* EOs in preservation of TSS content and increase of TAA and POD compared with untreated control (Table 6). The lower TSS content in this study indicated that EOs especially *R. officinalis* EO could postpone the process of strawberry fruit senescence during storage at 1°C. A similar result to Abdolahi et al. (2010), where the decrease in TSS content was attributed to reduce water loss in the fruits treated with EOs as observed in this study. Increase of TSS content during fruit storage might be occurred by the breakdown of the carbohydrates to simple sugars and degradation of polysaccharides in the fruit cell wall (Ben and Gaweda, 1985; Wills and Rigney, 2007; Ali et al., 2016) or organic acid consumption for respiration (Pelayo et al., 2003; Valero et al., 2006).

Generally, antioxidant activity in the fruits during postharvest periods may be reduced because of the degradation of cell structure, a consequence of fruit senescence (Macheix et al., 1990). In this study, the strawberry fruits treated with *A. sativum* and *R. officinalis* EOs significantly showed the higher TAA including POD compared with untreated control (Table 6). The protection of fruit cells from oxidative injury depends on the level of antioxidant enzymes (Petriccione et al., 2015). For example, antioxidant activity in plant cells can reduce membrane lipids peroxidation and protect cellular membranes of plant against plant pathogens (Franck et al., 2007). Peroxidase as one of the most important antioxidant enzymes regulates the metabolism of reactive oxygen species catalyzed by H_2_O_2_, a prerequisite for lignin synthesis to reinforce the cell wall and may also alter the antioxidant ability of fruit to cope with pathogens (Passardi et al., 2004). Both antioxidant and phenolic compounds in plant EOs have direct relationship in reduction of decay development in fruits during storage (Shen et al., 2013). It has been previously proven that phenolic compounds in plants treated with *Ocimum basilicum* increased the antioxidant activity and induced the defense system of plant against plant pathogens (Tzortzakis et al., 2011). Other studies indicated that EOs can induce the defense system of plants, through increasing the activity of defense-related enzymes and at least enhancing antioxidant capacity in plants (Ben-Jabeur et al., 2015; Khumalo et al., 2017).

Although a large number of studies are published on the composition of *A. sativum* and *R. officinalis* oils and their efficacy as pesticides, meta-analysis of these results is impossible because of inconsistency of methods and conditions used in the analysis (contact, vapor, concentration, time, environmental conditions at the application, etc.). Both *A. sativum* and *R. officinalis* EOs demonstrate in literature a wide spectrum of activity (insects, mites, fungi, bacteria), which can be considered an advantage in term of multiple-pest activity, but raises also the question of possible negative side effect on beneficial organisms and/or on human microbiota, which is for the two tested EOs still unknown.

Another important aspect in the practical feasibility of using EOs as alternatives to chemical pesticides is the availability of sufficient quantities at a reasonable cost for field application. The minimal inhibitory concentration or the effective concentrations found in literature and confirmed by our study indicate that they are in the order of few mg/L, which means an application dosage corresponding to several g/ha. In the case of *R. officinalis* EO in our study, the quantity of EO to be used per hectare is 680 mL, at a volume of application of 400 L/ha, and it corresponds to 68 kg of *R. officinalis* per hectare of treated strawberry field.

In current study, sensory evaluation revealed that EOs had no adverse effects on taste, odor, and flavors of fruit except those fruits, whereas their odor was affected by *A. sativum* EO. This result showed that apart from the positive effects of *A. sativum* on the reduction of the disease development, reduction or elimination of its negative effects on the fruit odor should be addressed in further studies. Sensory parameters such as taste, odor, and flavors of fruit are important factors, which can be changed by EOs and during storage time. Therefore, application of plant EOs against plant pathogens as biofungicide must not interfere with fruit sensorial quality and has to ensure their acceptability by consumers (Antunes et al., 2012; Sivakumar and Bautista-Banos, 2014; Vilaplana et al., 2018).

## Conclusion

In this study, the EOs of *A. sativum* and *R. officinalis* confirmed an expected promising fungicidal activity against *C. nymphaeae* constituting a potential source for the development of biofungicide. Moreover, EOs preserved the quality parameters in treated strawberry fruits. TP and POD of fruits increased in the fruit treated with *A. sativum* and *R. officinalis* EOs. Reduction of fruit decay development and DS in the fruits treated with the EOs of *A. sativum* and *R. officinalis* might be associated with an increase in phenol content and the activity of defense-related enzymes such as peroxidase. In addition, results showed that EOs were capable of maintaining the sensory quality such as fruit taste, flavor, and overall evaluation (except fruit odor) during the postharvest period. These results confirmed that both *A. sativum* and *R. officinalis* EOs can be utilized as biofungicides in the protection of strawberry instead of chemical fungicides against *C. nymphaeae*, with the lowest negative effects on the physicochemical, qualitative, and sensory properties of fresh strawberry fruits. However, the feasibility of use as fungicide must be discussed in a wider view before reaching the conclusion of having identified a promising alternative to chemical pesticides. In particular, the availability of sufficient quantities at a reasonable cost and the absence of negative effect on the quality of the treated fruits must be carefully considered in the development of a botanical fungicide.

## Data Availability Statement

The raw data supporting the conclusion of the results will be made available on request.

## Ethics Statement

The studies involving human participants were reviewed and approved by Research Ethics Committee of the University of Kurdistan in Iran. All participants provided written informed consent to participate in this study. The patients/participants provided their written informed consent to participate in this study.

## Author Contributions

SH performed the experiments and analyzed the data. JA and MS designed, analyzed, discussed the results, and wrote the manuscript. KK and IP discussed the results and wrote the manuscript. All authors contributed to the article and approved the submitted version.

## Conflict of Interest

The authors declare that the research was conducted in the absence of any commercial or financial relationships that could be construed as a potential conflict of interest.
